# Current understanding of adipose-derived mesenchymal stem cell-based therapies in liver diseases

**DOI:** 10.1186/s13287-019-1310-1

**Published:** 2019-07-08

**Authors:** Chenxia Hu, Lingfei Zhao, Lanjuan Li

**Affiliations:** Kidney Disease Center, First Affiliated Hospital, College of Medicine, Zhejiang University; Key Laboratory of Kidney Disease Prevention and Control Technology, Institute of Nephrology, Zhejiang University, Hangzhou, Zhejiang People’s Republic of China

## Abstract

The liver, the largest organ with multiple synthetic and secretory functions in mammals, consists of hepatocytes, cholangiocytes, hepatic stellate cells (HSCs), sinusoidal endothelial cells, Kupffer cells (KCs), and immune cells, among others. Various causative factors, including viral infection, toxins, autoimmune defects, and genetic disorders, can impair liver function and result in chronic liver disease or acute liver failure. Mesenchymal stem cells (MSCs) from various tissues have emerged as a potential candidate for cell transplantation to promote liver regeneration. Adipose-derived MSCs (ADMSCs) with high multi-lineage potential and self-renewal capacity have attracted great attention as a promising means of liver regeneration. The abundance source and minimally invasive procedure required to obtain ADMSCs makes them superior to bone marrow-derived MSCs (BMMSCs). In this review, we comprehensively analyze landmark studies that address the isolation, proliferation, and hepatogenic differentiation of ADMSCs and summarize the therapeutic effects of ADMSCs in animal models of liver diseases. We also discuss key points related to improving the hepatic differentiation of ADMSCs via exposure of the cells to cytokines and growth factors (GFs), extracellular matrix (ECM), and various physical parameters in in vitro culture. The optimization of culturing methods and of the transplantation route will contribute to the further application of ADMSCs in liver regeneration and help improve the survival rate of patients with liver diseases. To this end, ADMSCs provide a potential strategy in the field of liver regeneration for treating acute or chronic liver injury, thus ensuring the availability of ADMSCs for research, trial, and clinical applications in various liver diseases in the future.

## Introduction

The liver, the largest organ with multiple synthetic and secretory functions in mammals, consists of hepatocytes, cholangiocytes, hepatic stellate cells (HSCs), sinusoidal endothelial cells, Kupffer cells (KCs), and immune cells, among others [1]. Hepatocytes and cholangiocytes constitute the majority of liver parenchymal cells and play critical roles in maintaining liver function and biliary secretion; thus, the liver participates in the regulation of energy metabolism and detoxification. Under physiological conditions, HSCs, or fat-storing cells, are located in the parasinusoidal space; they mainly store retinoids and produce extracellular matrix (ECM) that is used in the generation of the basement membrane [2]. Liver sinusoidal endothelial cells are known to secrete several growth factors that promote hepatocyte proliferation, and they are responsible for forming new vasculature [3]. Liver KCs represent approximately 20% of the nonparenchymal cells in the liver and serve as an immune barrier for liver tissue; the activation of Kupffer cells acts as the priming force for hepatocyte proliferation [4]. Natural killer (NK) cells, natural killer T (NKT) cells, eosinophils, and other cells constitute the majority of cells associated with innate immunity in the liver and contribute to liver regeneration [5, 6]. Various causative factors, including viral infection, toxins, autoimmune defects, and genetic disorders, can impair liver function and result in chronic liver disease or acute liver failure. Although liver tissue has a remarkable ability to regenerate after injury, orthotopic liver transplantation (OLT) is still required to rescue patients with end-stage liver disease or liver failure involving large numbers of necrotic and apoptotic hepatocytes at the irreversible stage [7]. However, the application of OLT is limited by donor scarcity, the side effects of immunosuppressants, and ethical issues [8, 9]. A potential alternative to OLT, hepatocyte transplantation (HT), is simpler, less invasive, and safer; however, the application of HT is limited by the finite proliferation capacity and limited liver functions of primary hepatocytes [10]. Fortunately, mesenchymal stem cells (MSCs) from various tissues have emerged as potential candidates for cell transplantation to promote liver regeneration [11]. These multipotent cells are fibroblast-like and can differentiate into adipocytes, osteocytes, chondrocytes, hepatocytes, and other types of cells [12].

Bone marrow-derived MSCs (BMMSCs) have become the most common source of multipotent cells for transplantation in experimental studies and clinical trials since they were first isolated in 1970 by Friedenstein et al. [13]. To standardize MSCs, the International Society for Cell Therapy suggests the following minimal criteria [14]: adherence to plastic in conjunction with a fibroblastoid phenotype; expression of CD105, CD73, and CD90 and lack of expression of CD45, CD34, CD14 (or CD11b), CD79α (or CD19), and HLA-DR surface molecules; and the capacity to differentiate into chondrocyte, adipocyte, and osteocyte lineages. The low rate of immunological rejection of such cells makes it possible to use them in both autotransplantation and allogeneic transplantation applications [15]. MSCs have been reported to participate in repairing tissue or organ injury mainly through their paracrine effects, namely, stimulation of angiogenesis, protection of other cells from apoptosis, and recruitment of host MSCs or other progenitor cells and stimulation of their proliferation and differentiation [16]. MSCs also have anti-oxidative capacity that helps protect tissues against reactive oxygen species (ROS)-induced injury [17]. Moreover, cell fusion of MSCs also contributes to the repair of tissues and organ function [18]. These advantages allow MSCs to be used in the treatment of various diseases and to be clinically applied in the field of regenerative medicine.

The use of the iliac crest for bone marrow extraction is painful, and there is high risk of infection following this procedure [19]. Adipose-derived MSCs (ADMSCs) are collected from adipose tissue by liposuction, washing, collagenase digestion, and centrifugation in a process that is less invasive and easier than the harvesting of bone marrow cells; this permits wide use of ADMSCs [20]. The isolated stromal vascular fraction (SVF) of adipose tissue contains circulating blood cells, fibroblasts, pericytes, endothelial cells, and ADMSCs [21]. SVF is reported to contain 0.02 to 0.06% ADMSCs, whereas bone marrow mononuclear cells consist of only 0.001 to 0.01% BMMSCs [22]. The isolated undifferentiated ADMSCs express MSC surface markers and liver-specific genes including alpha fetoprotein (AFP), cytokeratin (CK)-18, CK-19, and hepatocyte nuclear factor (HNF)-4; moreover, they also weakly express albumin (ALB), glucose-6-phosphate, and α1-antitrypsin [23]. ADMSCs effectively maintain endothelial and vascular function via the secretion of vascular endothelial growth factor (VEGF) and nitric oxide (NO) [24, 25], and they exert an anti-oxidative effect via the upregulation of superoxide dismutase (SOD) and malondialdehyde (MDA) [26]. ADMSCs also participate in the stimulation of regulatory T cells (Tregs) and in the simultaneous suppression of Th1, Th2, and Th17 cells via the upregulation of immunomodulatory factors including IL-10, TGF-β, indolamine 2, and 3-dioxygenase and the downregulation of inflammatory factors such as IL-4, IL-12, IL-17, tumor necrosis factor (TNF)-α, interferon (IFN)-γ, t-bet, CD80, CD83, and CD86 [27, 28]. It is worth noting that IL-4 is primarily known for its anti-inflammatory effects due to its capacity to suppress Th1 responses and induce protective immunity against intracellular pathogens [29], while IL-4-producing Th2 cells directly mediate tissue destruction and can cause autoimmune disease if transferred to an immune-deficient host [30]. Intriguingly, ADMSCs were shown to survive for up to 4 months after transplantation in vivo [31]. Although ADMSCs share some of the biological properties of BMMSCs, they also have some distinct properties. For example, CD106, which is also known as vascular cell adhesion molecule 1 and is involved in cell migration, is expressed at significantly lower levels in ADMSCs than in BMMSCs [32]. On the other hand, both ADMSCs and BMMSCs express high levels of OCT4, NANOG, SOX2, alkaline phosphatase (ALP), and SSEA4 [33]. BMMSCs from aging donors demonstrated lower cell activity and differentiation capacities, whereas the cell activity of ADMSCs from aging donors is not limited [34, 35]. ADMSCs are superior in immune regulation compared to BMMSCs [36]; ADMSCs were shown to secrete higher levels of interleukin (IL)-6, IL-8, interleukin 1 receptor alpha (IL-1Rα), granulocyte colony-stimulating factor (G-CSF), granulocyte macrophage colony-stimulating factor (GM-CSF), monocyte chemotactic protein 1, nerve growth factor (NGF), and hepatocyte growth factor (HGF) than BMMSCs for elimination of liver injury [37]. Although ADMSCs secreted more NGF and transforming growth factor (TGF)-β1 than BMMSCs, they inhibited the proliferation and activation of HSCs to a comparable degree while promoting the apoptosis of HSCs for eliminating liver fibrosis [38]. In addition to a paracrine pathway, ADMSCs possess hepatogenic differentiation potential similar to that of BMMSCs as shown by their similar levels of expression of CK-18, CK-19, AFP, ALB, cytochrome (CYP), and other liver-enriched transcription factors but can be cultured for a longer period and have higher proliferation capacity [39, 40]. After transplantation in vivo into mice with acute liver failure (ALF), ADMSCs decreased the levels of alanine transaminase (ALT) and aspartate aminotransferase (AST) and improved liver histopathology more effectively than BMMSCs [41].

Given that ADMSCs are superior to BMMSCs in some respects, including ease of manipulation, abundance, and potentially higher stemness, herein we comprehensively analyze landmark studies of the isolation, proliferation, and hepatogenic differentiation of ADMSCs and summarize the therapeutic effects of ADMSCs in animal models with liver diseases. We also discuss key points for improving the hepatic differentiation of ADMSCs via exposure to cytokines and growth factors (GFs), extracellular matrix (ECM), and physical parameters in in vitro culture. The optimization of culturing methods and transplantation route will contribute to the further application of ADMSCs in liver regeneration and help improve the survival rate of patients with liver diseases in the near future.

### The source of ADMSCs

Adipose tissue can be collected from subcutaneous tissue [42], viscera [43], omentum [44], inguinal fat pads [45], peritoneal fat [46], and other sources. Although ADMSCs isolated from visceral adipose tissue appeared larger than those isolated from subcutaneous adipose tissue, both sets of ADMSCs showed similar pluripotency and plasticity and expressed MSC markers (CD105 and CD13) as well as other markers (SOX2, OCT4, LIF, and NANOG) [43]. ADMSCs isolated from human liver falciform ligaments showed higher levels of hematopoietic- and mesenchymal-epithelial transition (MET)-related surface markers than ADMSCs obtained from human abdominal subcutaneous adipose tissue, whereas both groups of cells display similar proliferation, multi-lineage capacity, and hepatic induction [47]. Considering that ADMSCs from visceral and subcutaneous tissues are comparable in pluripotency, plasticity, and hepatogenic differentiation, the ease of acquisition currently makes subcutaneous adipose tissue the optimal source of ADMSCs.

Allogeneic ADMSCs are isolated from a cell donor other than the cell recipient, while autologous ADMSCs are isolated from the cell recipient. Autologous ADMSCs serve as the ideal source since their use involves no ethical issues and they display high histocompatibility and low immune rejection [48]. Strong et al. demonstrated that ADMSCs isolated from animals with chronic inflammatory diseases such as obesity and multiple sclerosis were less effective in immunomodulation [49], while Hu et al. demonstrated that ADMSCs isolated from ALF pigs have stem cell characteristics and cell activities similar to those of ADMSCs from control pigs; however, ADMSCs from ALF pigs showed increased expression of several liver-specific genes [50]. Although BMMSCs from patients with chronic hepatitis B infection proliferated poorly and were limited to hepatogenic differentiation, ADMSCs from these patients were not susceptible to infection by hepatitis B virus [51]. These findings indicate that allogeneic ADMSCs can be used in the treatment of patients with liver diseases.

Although the cellular phenotype and level of apoptosis displayed by ADMSCs obtained from infants, adults, and elderly people are similar, ADMSCs isolated from infants display a higher capacity for proliferation and migration. ADMSCs derived from adults and elderly people were significantly less efficient at suppressing T cell proliferation and showed increased production of IFN-γ and decreased production of IL-10 compared with infant-derived ADSCs, indicating that an age-associated decline in the immunomodulatory capacity of ADMSCs occurs [52]. Sequential passage in vitro exerts a negative impact on the multipotency of ADMSCs [53], and long-term culture results in replicative senescence, genetic instability, and upregulated immune responses in ADMSCs and consequently reduces their therapeutic efficacy [54, 55]. Thus, ADMSCs isolated from infants or early-passage cells may have greater potential to be effective in promoting liver regeneration than ADMSCs obtained from adults and elderly people and late-passage ADMSCs.

### Hepatogenic differentiation in vitro and application of HLCs in vivo

#### Hepatogenic differentiation in vitro

ADMSCs are easily differentiated into hepatocyte-like cells (HLCs) as they change in morphology and cell function after treatment with specific cytokines and when exposed to a liver-damaged internal microenvironment [56]. ADMSC-derived HLCs exhibit several liver-specific functions, including ALB secretion, glycogen synthesis, urea formation, low-density lipoprotein uptake, CYP enzyme activity, and expression of carbamoylphosphate synthetase [11, 57]. HLCs derived from ADMSCs express periportal functions, including carbamoylphosphate synthetase 1 and the entry enzyme of the urea cycle, as well as perivenous functions, including CYP450 subtype 3a11 and CD26 [58]. Furthermore, the gene expression profiles of HLCs reveal a striking similarity between HLCs and liver tissue in their gene clusters, genes, and signaling pathways and MET transition [59]. ADMSCs can be induced to differentiate into hepatocytes by culturing for 2 weeks in hepatogenic medium containing dexamethasone, insulin, HGF, and epidermal growth factor (EGF); the ADMSCs then complete the hepatogenic differentiation process via activation of the extracellular signal-regulated kinase (ERK)/mitogen-activated protein kinase (MAPK) signaling pathway [56]. Step-by-step hepatogenic differentiation of MSCs promotes the generation of HLCs, as demonstrated by the appearance of early markers (ALB, alpha-2-macroglobulin, complement protein C3, and selenoprotein P1) and late markers (CYP, apolipoprotein E, acyl-CoA synthetase long-chain family member 1, and angiotensin II receptor, type 1). The loss of stem cell phenotype by these cells was detected by loss of expression of THY1 and inhibitor of DNA binding 3 [60].

Although current studies use various types of differentiation protocols, ADMSC-derived HLCs have immature hepatocyte functions; thus, specialists have attempted to develop new methods to improve the functions of HLCs. Serum from rats that underwent 70% partial hepatectomy (PH) promoted the hepatogenic differentiation of ADMSCs in vitro by upregulating the secretion of IL-6 and HGF [61]. In addition, ADMSCs exhibited more rapid changes in cellular morphology and expressed higher levels of AFP and ALB after incubation with liver extract than after culture in the presence of chemicals including HGF, fibroblast growth factor (FGF), and oncostatin M [62]. Trichostatin A, a specific histone deacetylase inhibitor, significantly enhanced the hepatogenic differentiation of ADMSCs by upregulating the expression of miR-122, ALB, HNF4α, and HNF6 while downregulating the AFP level [63]. Dimethyl sulfoxide, a common cryoprotectant, accelerated the hepatic differentiation of ADMSCs as shown by rapid changes in cell morphology, increased expression of ALB, CK18, HNF4α, and HNF6 and greater glycogen storage in the differentiated ADMSCs [64]. After incubation with activin A and FGF4 for 3 days and subsequent incubation with HGF, FGF1, FGF4, oncostatin M, dexamethasone, insulin–transferrin–selenium, dimethyl sulfoxide, and nicotinamide for 10 days, ADMSCs acquired the functional properties of primary human hepatocytes in vitro [65]. Using a three-step protocol involving incubation with IDE1 and CHIR99021; incubation with IDE1, FGF4, and HGF; and a final step that included exposure of the cells to HGF, EGF, oncostatin M, dexamethasone, and insulin–transferrin–selenium, Xu et al. induced ADMSCs to transform into HLCs with the functions of mature hepatocytes within 9 days [66]. In addition to culture in hepatic medium, gene modification also contributes to promotion of the hepatogenic differentiation of ADMSCs. Overexpression of OCT4 and SOX2 did not alter the expression of MSC markers or morphology in ADMSCs but did enhance the expression of ALB, urea, and glycogen in hepatogenic ADMSCs [67]. MicroRNAs (miRNAs) are small noncoding RNAs that help regulate diverse biological processes such as metabolism, proliferation, the cell cycle, and differentiation. The possible mechanism through which this occurs may be microRNA-mediated expression of GFs and cytokines, as miR-122 and miR-27b have been reported to play a critical role in the hepatogenic differentiation of ADMSCs [68, 69]. ADMSCs can be differentiated into HLCs by stable miR-122 overexpression and let-7f silencing without other stimulation. These genetically modified ADMSCs showed significantly increased expression of hepatocyte markers including ALB, AFP, CK-18, CK-19, and HNF-4a and upregulated urea, ALB, and glycogen production [70].

**Fig. 1:**
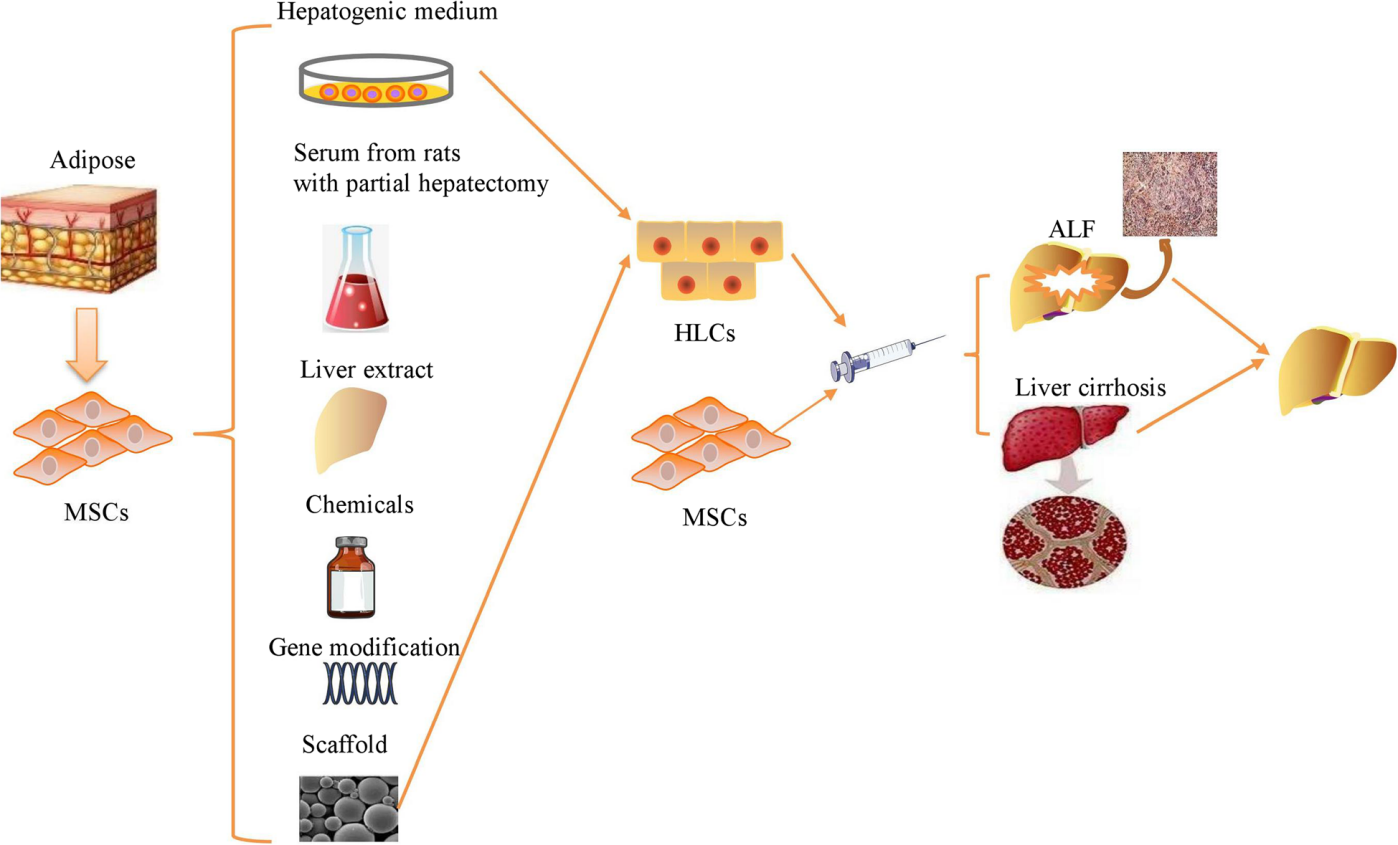
Transplantation of HLCs and ADMSCs contributes to liver regeneration in various liver diseases

In recent years, the biochemical and mechanical signals provided by the ECM have been shown to effectively enhance the proliferation and differentiation of ADMSCs. When cultured on spots containing HGF and collagen I, ADMSCs showed significantly upregulated expression of ALB, AFP, and α1-antitrypsin compared to ADMSCs cultured on spots containing only collagen I [71]. Fabricated gelatin scaffolds with high biocompatibility promoted the adhesion and proliferation of ADMSCs without any adverse effects and significantly enhanced the hepatogenic differentiation of ADMSCs compared to culture on two-dimensional tissue culture polystyrene [72]. Furthermore, ADMSCs cultured on a three-dimensional scaffold consisting of gelatin cryogel and laminin displayed increased attachment and improved liver functions similar to those of HepG2 cells [73]. In the presence or absence of GFs, a liver decellularized matrix enhanced the hepatic differentiation of ADMSCs into mature hepatocytes significantly more effectively than other coating matrices including collagen, fibronectin, and Matrigel [74]. The ultimate aim of in vitro hepatogenic differentiation of ADMSCs is the acquisition of functional mature hepatocytes for HT in vivo; the safety of using modified cell culture microenvironments and of using the ADMSCs themselves should also be a matter of concern (Fig. 1).

#### Application of ADMSC-derived HLCs in vivo

Transplantation of HLCs before ischemia ameliorated hepatic dysfunction and improved liver regeneration after extended resection-induced ALF via attenuation of metabolic overload and normalization of amino acid, acylcarnitine, sphingolipid, and glycerophospholipid levels [46]. HLCs also reduced the levels of expression of ALT, AST, and ammonia and restored liver functions, including ammonia and purine metabolism, in ALF mice [65]. These ADMSC-derived HLCs showed more consistent gene expression and a more normal hepatogenic differentiation profile than HLCs from BMMSCs; moreover, transplantation of ADMSCs, BMMSCs, and HLCs derived from ADMSCs and of HLCs derived from BMMSCs promoted liver regeneration in carbon tetrachloride (CCl_4_)-induced ALF mice to comparable degrees [75]. However, there is a debate concerning the use of ADMSCs and HLCs in vivo. As Guo et al. demonstrated, transplantation of ADMSCs and HLCs improved liver function and rescued CCl_4_-treated mice with liver injury, but ADMSC transplantation improved liver functions more effectively than transplantation of HLCs [76]. HLCs significantly restored liver function and prolonged the survival of mice with CCl_4_-induced ALF by engraftment into the injured liver, but infusion of the liver with primary hepatocytes was not effective [66]. Furthermore, transplantation of HLCs eliminated CCl_4_-induced liver fibrosis and preserved liver functions via the secretion of TGF-β1, IL-6, and IL-10 [77]. Engineered hepatic grafts that combined acellular human amniotic membrane with HLCs derived from ADMSCs significantly decreased the degree of CCl_4_-induced liver injury by improving the expression of ALB, HNF-4α, and CYP450 2B6 [78]. However, Bruckner et al. demonstrated that these HLCs decreased the amount of collagen, the portal venous pressure, and the splenic weight but had no effect on the improvement of liver dysfunction, fibrillary collagen content, the balance of matrix metalloproteinases (MMPs) and metalloproteinases (TIMPs), or the activation of HSCs [79]. To this end, hepatogenic ADMSCs can be used in the treatment of various liver diseases, but future studies should further investigate the potential mechanisms through which HLCs function in liver regeneration. The therapeutic effects of HLCs derived from ADMSCs can then be further improved for application in experimental and clinical trials.

### ADMSC transplantation for liver regeneration

**Fig. 2:**
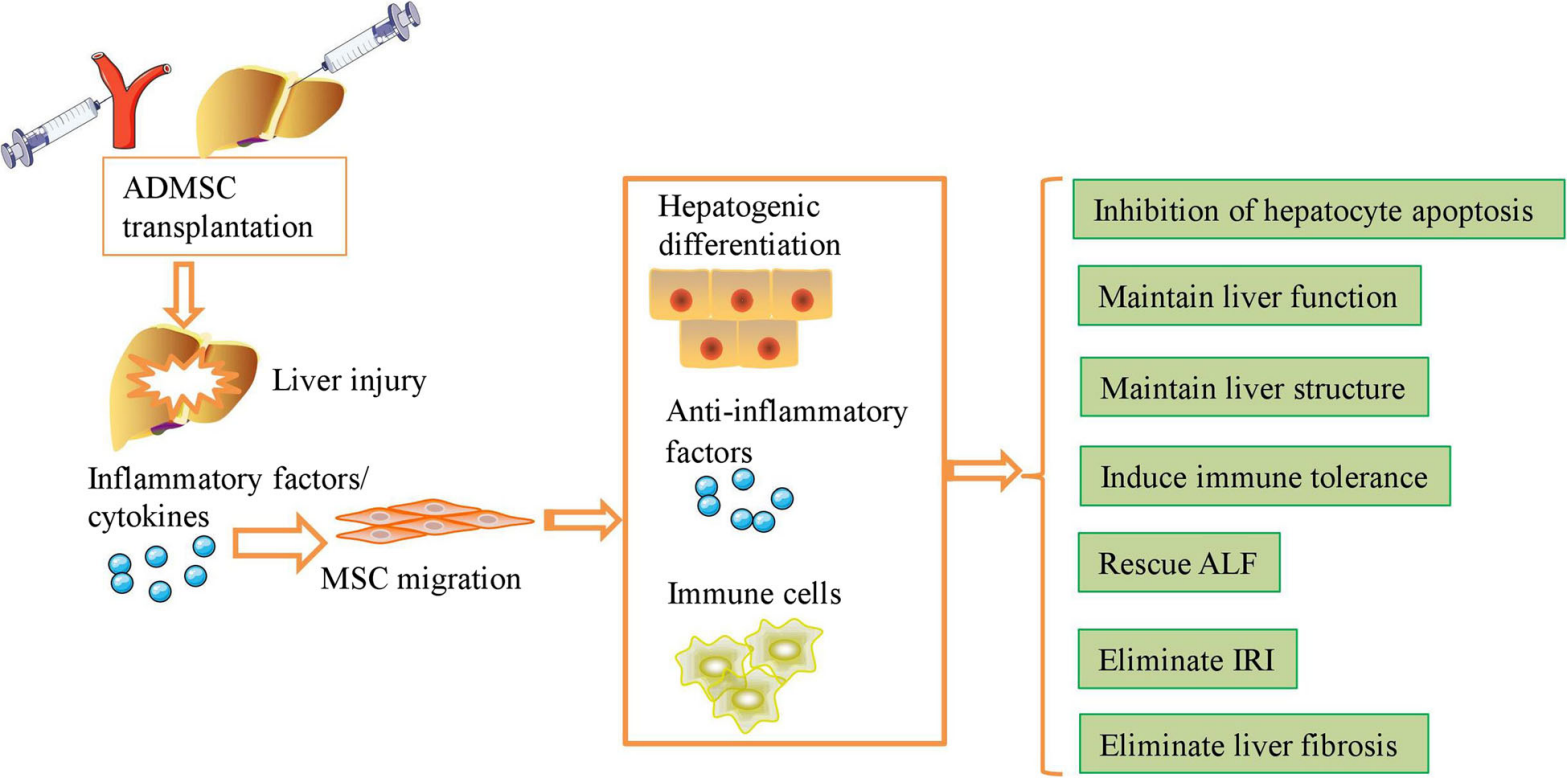
ADMSCs engraft in vivo and repair injured liver tissue via differentiation, immunomodulatory effects, and paracrine effects

ADMSCs engraft in vivo and repair injured tissue via differentiation, immunomodulatory effects, and paracrine effects [80] (Fig. 2). Injured liver tissue and hepatocytes secrete various inflammatory factors and chemotactic cytokines that attract ADMSCs to the site of injury. ADMSCs are reported to produce tonofilaments and to then enter the injured sites after activation of the stromal-derived factor-1 (SDF-1)/C-X-C chemokine receptor type 4 (CXCR4) axis in the injured liver [81]. Furthermore, engrafted ADMSCs secrete various cytokines, including HGF and FGF, that promote the regeneration of endogenous hepatocytes and thereby help maintain the normal structure of the liver [82, 83]. ADMSC transplantation significantly increased regeneration of the remaining liver following repeat PH, as demonstrated by upregulation of the liver-to-body-weight ratio, HGF, and PCNA levels and downregulation of aminotransferases, total bilirubin (TBIL), and hepatic vacuolar degeneration at 24 h post-hepatectomy; moreover, the liver showed complete recovery at 168 h after ADMSC transplantation [84]. MiR-27b-overexpressing ADMSCs enhanced liver regeneration and preserved hepatic function via the downregulation of inflammatory cytokines and the upregulation of HGF, HO-1, and mitochondrial biogenesis in a PGC-1α-dependent manner in PH rats [85].

Intravenously injected ADMSCs engrafted into various tissues, including brain, thymus, heart, liver, and lung, while PH enhanced the integration of ADMSCs into the liver and increased the regeneration of injured liver [86]. Transplantation of ADMSCs via the tail vein reduced biochemical parameters such as ALT, AST, and ammonia in CCl_4_-induced liver injury more effectively than transplantation via the portal vein or direct liver parenchymal injection [87]. Transplantation of ADMSCs via the peripheral vein or the splenic vein decreased the levels of proinflammatory cytokines, including IL-1, IL-6, IL-8, and IFN-*γ*, and increased the levels of anti-inflammatory cytokines, including IL-4 and IL-10, HGF, and VEGF in an ALF animal model, while transplantation via the splenic vein significantly decreased the levels of serum liver enzymes and increased the number of engrafted ADMSCs in the liver more effectively than transplantation via the peripheral vein [88]. Wang et al. reported that administration of ADMSCs via the portal vein significantly decreased the hepatic arterial perfusion index but increased portal vein perfusion and microcirculation in rats with liver fibrosis [89]. According to current evidence regarding transplantation route, transplantation via the peripheral vein appears to be the most convenient method, but determination of which route is the most effective requires further study.

#### Ischemia/reperfusion-induced injury

Ischemia-reperfusion injury (IRI) of the liver is a well-known cause of morbidity and mortality after OLT and HT. ADMSCs decreased the apoptosis of hepatocytes, decreased the levels of ALT, AST, TBIL, IL-2, and IL-10, and maintained the tissue structure in rats with OLT via alleviation of acute rejection [90]. ADMSCs improved the survival rate of rats with liver IRI by downregulating IL-6, IL-21, and CD70 and activating the neurogenic locus Notch homolog protein pathway; the necrotic areas showed improved liver function and improved liver regeneration and maintained normal histology [91, 92]. Intrahepatic transplantation of ADMSCs markedly reduced the apoptosis of hepatocytes and decreased the severity of pathological changes via downregulation of Fas, Fas ligand, caspase-3, caspase-8, and caspase-9 and upregulation of the Bcl-2/Bax ratio in pigs with IR combined with laparoscopic hepatectomy [93]. ADMSCs significantly decreased the serum levels of ALT, AST, TBIL, and lactate dehydrogenase (LDH) via upregulation of SOD, suppression of myeloperoxidase (MPO) and MDA, and suppression of autophagy in swine with IRI [94]. On the other hand, administration of ADMSCs decreased hepatic oxidative stress and the expression of TNF-α, TGF-β, IL-1β, IL-6, endothelin-1, MMP-9, plasminogen activator inhibitor-1, Bax, caspase-3, and intercellular adhesion molecule but increased the levels of endothelial nitric oxide synthase, Bcl-2, IL-10, quinone oxidoreductase 1, and HO-1 in liver with IRI [95]. Sudden and prolonged interruption of the arterial blood flow to the liver accompanied by reperfusion initiated oxygen and nutrient deprivation, upregulation of oxidative reactions, and activation of inflammation in the liver, while ADMSCs are effective in eliminating IRI in liver tissues.

#### Chemically induced acute liver injury

As we know, the liver is the first organ to come into contact with various orally ingested drugs after intestinal absorption; thus, it is susceptible to chemically induced injury, and such injury can result in acute and chronic liver disease [96]. Banas et al. showed that ADMSC transplantation markedly improved liver functions and maintained the levels of ammonia, uric acid, and transaminases in animals with CCl_4_-induced injury [37]. Animals treated with ADMSCs prior to CCl_4_-induced ALF also demonstrated lower levels of ALT and IL-6 and higher expression of regeneration markers, accompanied by improved histopathology and survival rate [97]. In addition, spheroid-derived ADMSCs significantly increased liver regeneration in mice with CCl_4_-induced ALF compared to ADMSCs derived from constant monolayer cultures [98], and regenerated silk fibroin (RSF)-treated ADMSCs rescued CCl_4_-induced ALF animals via upregulation of angiogenesis and hepatogenic differentiation more effectively than ADMSCs on neat RSF scaffolds [99].

On the other hand, ADMSCs significantly decreased the levels of ALT, AST, and ammonia and returned prothrombin time to normal levels in acetaminophen (APAP)-induced ALF rats via inhibition of liver stress and inflammatory signaling and enhancement of liver regeneration [42]. In addition, ADMSC transplantation significantly attenuated the severity of APAP-induced liver injury and improved the survival rate of APAP-induced ALF mice via suppressing MAPK signal activation, reducing the level of toxic nitrotyrosine and upregulating NF-E2-related factor 2 (Nrf2) expression and anti-oxidant activity [44]. The immunomodulatory effect of ADMSCs may serve as an important mechanism in enhancing liver regeneration and maintaining liver histology without necrosis in the livers of mice with concanavalin A (ConA)-induced hepatitis. Kubo et al. demonstrated that ADMSCs significantly downregulated the levels of liver enzymes, decreased the histopathological changes and increased the survival rate of mice with ConA-induced fulminant hepatitis via suppression of inflammatory cytokines and a reduction in the number of CD11b^+^, Gr-1^+^, and F4/80^+^ cells [100–102].

To improve the therapeutic effects of ADMSCs in vivo, preconditioning with lysophosphatidic acid (LPA) and/or sphingosine-1-phosphate (S1P) has been used. Treatment of cells with these agents synergistically enhanced the anti-stress effects of ADMSCs via Gi protein, the RAS/ERK pathway, the PI3K/AKT pathway, upregulation of IL-10, and promotion of the nuclear translocation of nuclear factor-kappa B (NF-κB). These LPA- and/or S1P-pretreated ADMSCs obviously ameliorated the histological damage, oxidative stress, inflammation, and lipid metabolism dysfunction in galactoside (Gal)/lipopolysaccharide (LPS)-induced ALF mice [103]. Although zeaxanthin dipalmitate (ZD)-pretreated ADMSCs exerted no adverse effects on healthy animals, they significantly improved liver function in a Gal/LPS-induced ALF model via upregulation of microRNA-210 and subsequent suppression of apoptosis, inflammation, and ROS in ADMSCs [104].

#### Liver fibrosis

Sustained hepatitis virus infection, alcohol consumption, and fat deposition lead to repeated and chronic liver injury, and the resulting accumulation of aberrant myofibroblasts and extracellular matrix results in liver fibrosis with poor prognosis. ADMSC transplantation significantly reduced serum levels of glutamic pyruvate transaminase and TBIL and reduced liver fibrosis as evidenced by Sirius Red staining [105]. Harm et al. concluded that the detailed mechanism through which ADMSCs eliminate liver fibrosis involves hepatic differentiation, reduction of inflammatory activity, and inhibition of HSC activation [106]. Furthermore, ADMSCs significantly reduced the expression of collagen I, collagen III, α-smooth muscle actin (α-SMA), hyaluronic acid, and hydroxyproline and inhibited liver fibrogenesis via inhibition of the activation of HSCs, enhancement of HSC apoptosis, upregulation of HGF, and downregulation of NGF and TGF-β1 [107]. A clinical study enrolled four patients with liver cirrhosis for ADMSC transplantation. The study found that ADMSCs maintained liver function well and that they upregulated the expression of liver regeneration-related factors (HGF and IL-6) in patients with liver cirrhosis [108].

Splenectomy prior to MSC administration suppressed liver fibrosis via upregulation of stromal cell-derived factor-1 and HGF, which facilitate the migration of MSCs into injured sites [109]. After incubation with serum from rats with acute CCl_4_ injury, ADMSCs demonstrated polygonal morphology and expressed AFP, ALB, and CK8 and other hepatocyte markers. Moreover, ADMSCs preconditioned with serum from rats with acute CCl_4_ injury significantly improved liver function and reduced liver fibrosis in CCl_4_-induced liver fibrosis, as demonstrated by higher expression of hepatic and pro-survival markers and improvement in liver structure [110]. Exposure to basic fibroblast growth factor obviously upregulated the proliferation and differentiation of ADMSCs in vitro and enhanced the ability of ADMSCs to suppress the progression of liver fibrosis via elevation of HGF expression, promotion of HSC apoptosis, and enhancement of hepatocyte proliferation [83].

Genetically modified ADMSCs are currently being used in the treatment of liver fibrosis since they are able to accelerate repair of liver injury in vivo. HGF-overexpressing ADMSCs significantly decreased the serum levels of ALT and AST, ameliorated radiation-induced liver fibrosis via downregulation of α-SMA and fibronectin, and promoted hepatocyte regeneration [82]. Transplantation of FGF-21-overexpressing ADMSCs significantly attenuated thioacetamide-induced liver fibrosis via inhibition of p-JNK, NF-κB, and p-Smad2/3 signaling and secretion of α-lactoalbumin and lactotransferrin [111]. Overexpression of miR-122 enhanced the therapeutic efficacy of ADMSCs by suppressing proliferation and collagen maturation in HSCs in the treatment of CCl_4_-induced liver fibrosis [112].

## Conclusions

In comparison to MSCs from other sources, ADMSCs have similar multi-lineage potential, self-renewal capacity, anti-apoptotic effects, anti-oxidative effects, and anti-inflammatory effects after administration in vivo. We suggest that the application of ADMSCs in liver regeneration be increased since they have unique characteristics such as abundant source material and ease of isolation. Although various studies have focused on improving the hepatic functions of HLCs in vitro, these immature hepatocytes easily progress to the cell death pathway. Thus, we still recommend implanting ADMSCs in vivo since they are not extremely sensitive to the damaged microenvironment. Moreover, autologous ADMSCs are recommended because the use of autologous cells reduces the acute rejection rate. The liposuction process causes less trauma than bone marrow aspiration, and adipose tissue can provide a large number of ADMSCs for proliferation and injection. The ideal route of administration, dosage, and timing of ADMSC administration for the treatment of liver disease are highly variable; thus, studies should focus on the optimization of ADMSC efficacy in vivo. However, the short-term and long-term safety of the clinical application of cell transplantation is also an area of active controversy as cell transplantation may result in infections and potentially in tumorigenesis. To this end, ADMSCs provide a potential strategy in the field of liver regeneration for treating acute or chronic liver injury (Table 1), thus ensuring the availability of ADMSCs for research, trial, and clinical applications in various liver diseases in the future.

**Table 1:** ADMSCs effectively participate in liver regeneration in the treatment of various liver diseases

Dose	Route	Pretreatment	IR method	Animal	Effect	Mechanism	Ref.
2 × 10^6^	Tail vein	N/A	Liver transplantation	Rat	Decrease the apoptosis of hepatocytes; decrease the levels of ALT, AST, and TBIL; maintain the tissue structure	Decrease the expression of IL-2 and IL-10	[90]
1 × 10^5^	Jugular vein	N/A	70% partial hepatectomy	Mice	Liver regeneration	Integration of ADMSCs into the liver	[86]
1-2 × 10^6^	Tail vein	Before ischemia	70% partial hepatectomy	Mice	Improve histopathological changes; decrease serum levels of hepatocyte markers	Enhance hepatocyte proliferation	[91]
4 × 10^6^	Liver lobe	N/A	Bulldog clamp for 90 min and clamp removal	Rats	Decrease necrotic areas and improve liver function	Suppress the proinflammatory cytokines IL-6, IL-21, and CD70; activate the NOTCH Pathway	[92]
2 × 10^6^	Portal vein	N/A	Repeated partial hepatectomy	Rats	Increase body weight ratio; improve liver function; improve PCNA-labeling index	Upregulate expression of HGF	[84]
1 × 10^6^/kg	Liver parenchyma	N/A	Partial hepatectomy	Pigs	Reduce pathological and ultrastructural changes and decrease the number of apoptosis-positive cells	Downregulate the expression of Fas, Fas ligand, caspase-3, caspase-8, and caspase-9; upregulate of the ratio of Bcl-2/Bax	[93]
					Decrease the serum levels of ALT, AST, TBIL, and LDH	Upregulate the expression of SOD; suppress the expression of MPO and MDA; suppress autophagy	[94]
1.2 × 10^6^	Liver lobe	N/A	Occlude the vascular supply of the left lobe of the liver for 60 m followed by reperfusion for 72 h	Rats	Reduce plasma aminotransferases; promote liver regeneration	Suppress cellular activation; reduce proinflammatory cytokine release; alleviate oxidative stress; preserve hepatic microcirculation; decrease apoptosis	[95]
2 × 10^6^	Intravenous	Overexpression of MiR-27b	PH	Rats	Enhance liver regeneration and preserve hepatic function	Downregulate the expression of inflammatory cytokines; upregulate the expression of HGF, HO-1, and mitochondrial biogenesis in a PGC-1α-dependent manner	[85]
1–2 × 10^6^	Tail vein	Before CCl_4_	CCl_4_	Mice	Decrease levels of ALT and serum IL-6; increase the expression of regeneration markers and PCNA; improve histopathology; improve survival rate	Inhibit inflammation and liver necrosis	[97]
4 × 107 cells/kg	Spleen	Spheroid	CCl_4_	Mice	Increase liver regeneration	Inhibit hepatocyte necrosis	[98]
1 × 10^6^	Liver lobe	RSF	CCl_4_	Mice	Increase the survival rate of ALF animals	Upregulate angiogenesis and hepatogenic differentiation	[99]
1.0 × 10^6^	Intravenously	N/A	Con A	Mice	Increase the survival rate of ConA-induced fulminant hepatitis mice; decrease liver enzyme levels; improve histopathological changes	Suppress inflammatory cytokines	[100]
1 × 10^5^	Tail vein	N/A	Con A	Mice	Liver histology showed an almost normal appearance, with no necrosis	Repress inflammatory cell accumulation	[101]
1.0 × 10^6^	Tail vein	N/A	Con A	Mice	Decrease liver enzyme levels; improve histopathological changes	Decrease inflammation related to IL-6, IL-10, IFN-γ, and TNF-α	[102]
2 × 10^6^	Tail vein	LPA and/or S1P	Gal/LPS	Mice	Enhance survival rate of Gal/LPS-induced ALF mice; ameliorate histological damage;	Reduce oxidative stress, inflammation and lipid metabolism dysfunction	[103]
2 × 10^6^	Tail vein	ZD	Gal/LPS	Mice	Improve liver function of ALF model; exert no adverse effects on healthy animals	Activation of the PKC/Raf-1/MAPK/NF-κB pathway; upregulate microRNA-210	[104]
1.0 × 10^6^	Tail vein	N/A	CCl_4_	Mice	Reduce serum levels of glutamic pyruvate transaminase and TBIL; reduce hepatocyte vacuolar degeneration; decrease serum transaminase levels; inhibit liver fibrogenesis	Increase MMP-3 and MMP-9 levels	[105]
1.0 × 10^6^	Liver lobe	N/A	Thioacetamide	Rats	Eliminate liver fibrosis	Hepatic differentiation; reduce inflammation and inhibit HSC activation	[106]
5 × 10^6^	Portal vein	N/A	CCl_4_	Rats	Reduce the fibrotic area; reduce the expression of collagen I and a-SMA in the liver; reduce hydroxyproline level in the live; reduce collagen III and hyaluronic acid levels; inhibit liver fibrogenesis	Inhibit the proliferation and activation of HSCs; enhance HSC apoptosis; increase HGF level; decrease levels of NGF and TGF-b1	[107]
3 × 10^5^/kg and 6.6 × 10^5^/kg	Hepatic artery	N/A	Cirrhosis	Patients	Improve liver function	Increase serum HGF and IL-6 concentrations	[108]
5 × 10^6^	Caudal vein	Splenectomy	CCl_4_	Rats	Improve liver function; reduce levels of α-SMA and TGF-β; suppress liver fibrosis	Upregulate the levels of stromal cell-derived factor 1 and HGF; enhance the migration of ADMSCs into injured sites; promotes HSC apoptosis	[109]
1.5 × 10^6^	Intrahepatic	Serum from acute CCl4 injury rat	CCl_4_	Rats	Improve liver functions; reduce liver fibrosis	Increase the homing of ADMSCs	[110]
1.5 × 10^6^	Tail vein	Overexpression of FGF21	Thioacetamide	Mice	Decrease serum hyaluronic acid; reduce serum ALT, AST, and hyaluronic acid levels; reduce expression of fibrosis-related factors such as α-SMA, collagen and TIMP-1;	Inhibition of p-JNK, NF-κB, and p-Smad2/3 signaling and secretion of LA and LTF	[111]
1 × 10^5^	Tail vein	Overexpression of MiR-122	CCl_4_	Mice	Decrease serum levels of ALT, AST, and liver hydroxyproline content; reduce mature Col1A1 protein level	Suppress the proliferation of and collagen maturation in HSCs; decrease the expression levels of TGF-β1 and α-SMA in the liver	[112]

## Data Availability

All data are included in this published article.

## Abbreviations

ADMSCs: Adipose-derived MSCs
AFP: Alpha fetoprotein
ALB: Albumin
ALF: Acute liver failure
ALP: Alkaline phosphatase
ALT: Alanine transaminase
APAP: Acetaminophen
AST: Aspartate aminotransferase
BMMSCs: Bone marrow-derived MSCs
CCl4: Carbon tetrachloride
CK: Cytokeratin
ConA: Concanavalin A
CXCR4: C-X-C chemokine receptor type 4
CYP: Cytochrome
ECM: Extracellular matrix
ERK: Extracellular signal-regulated kinase
FGF: Fibroblast growth factor
Gal: Galactoside
G-CSF: Granulocyte colony-stimulating factor
GFs: Growth factors
GM-CSF: Granulocyte macrophage colony-stimulating factor
HGF: Hepatocyte growth factor
HLCs: Hepatocyte-like cells
HNF: Hepatocyte nuclear factor
HSCs: Hepatic stellate cells
HT: Hepatocyte transplantation
IFN: Interferon
IL: Interleukin
IL-1Rα: Interleukin 1 receptor alpha
IRI: Ischemia-reperfusion injury
KCs: Kupffer cells
LDH: Lactate dehydrogenase
LPA: Lysophosphatidic acid
LPS: Lipopolysaccharide
MAPK: Mitogen-activated protein kinase
MDA: Malondialdehyde
MET: Mesenchymal-epithelial transition
miRNAs: MicroRNAs
MMPs: Matrix metalloproteinases
MPO: Myeloperoxidase
MSCs: Mesenchymal stem cells
NF-κB: Nuclear factor-kappa B
NGF: Nerve growth factor
NK: Natural killer
NKT: Natural killer T
NO: Nitric oxide
Nrf2: NF-E2-related factor 2
OLT: Orthotopic liver transplantation
PH: Partial hepatectomy
ROS: Reactive oxygen species
RSF: Regenerated silk fibroin
S1P: Sphingosine-1-phosphate
SDF-1: Stromal-derived factor-1
SOD: Superoxide dismutase
SVF: Stromal vascular fraction
TGF: Transforming growth factor
TIMPs: Metalloproteinases
TNF: Tumor necrosis factor
Tregs: Regulatory T cells
VEGF: Vascular endothelial growth factor
ZD: Zeaxanthin dipalmitate
α-SMA: α-smooth muscle actin

## References

[1] Yang X, He C, Zhu L, Zhao W, Li S, Xia C, Xu C (2019). Comparative analysis of regulatory role of Notch signaling pathway in 8 types liver cell during liver regeneration. Biochem Genet.

[2] D'Ambrosio DN, Walewski JL, Clugston RD, Berk PD, Rippe RA, Blaner WS (2011). Distinct populations of hepatic stellate cells in the mouse liver have different capacities for retinoid and lipid storage. PLoS One.

[3] Ding BS, Nolan DJ, Butler JM, James D, Babazadeh AO, Rosenwaks Z, Mittal V, Kobayashi H, Shido K, Lyden D, Sato TN, Rabbany SY, Rafii S (2010). Inductive angiocrine signals from sinusoidal endothelium are required for liver regeneration. Nature..

[4] Fernandez V, Reyes S, Bravo S, Sepulveda R, Romanque P, Santander G, Castillo I, Varela P, Tapia G, Videla LA (2007). Involvement of Kupffer cell-dependent signaling in T3-induced hepatocyte proliferation in vivo. Biol Chem.

[5] Dong Z, Wei H, Sun R, Tian Z (2007). The roles of innate immune cells in liver injury and regeneration. Cell Mol Immunol.

[6] Goh YP, Henderson NC, Heredia JE, Red Eagle A, Odegaard JI, Lehwald N, Nguyen KD, Sheppard D, Mukundan L, Locksley RM, Chawla A (2013). Eosinophils secrete IL-4 to facilitate liver regeneration. Proc Natl Acad Sci U S A.

[7] Miro JM, Laguno M, Moreno A, Rimola A (2006). Management of end stage liver disease (ESLD): what is the current role of orthotopic liver transplantation (OLT)?. J Hepatol.

[8] Routh D, Naidu S, Sharma S, Ranjan P, Godara R (2013). Changing pattern of donor selection criteria in deceased donor liver transplant: a review of literature. J Clin Exp Hepatol.

[9] Dutkowski P, Clavien PA (2014). Solutions to shortage of liver grafts for transplantation. Br J Surg.

[10] Forbes SJ, Alison MR (2014). Regenerative medicine. Knocking on the door to successful hepatocyte transplantation. Nat Rev Gastroenterol Hepatol.

[11] Aurich H, Sgodda M, Kaltwasser P, Vetter M, Weise A, Liehr T, Brulport M, Hengstler JG, Dollinger MM, Fleig WE, Christ B (2009). Hepatocyte differentiation of mesenchymal stem cells from human adipose tissue in vitro promotes hepatic integration in vivo. Gut..

[12] Hu C, Li L (2015). In vitro culture of isolated primary hepatocytes and stem cell-derived hepatocyte-like cells for liver regeneration. Protein Cell.

[13] Friedenstein AJ, Chailakhjan RK, Lalykina KS (1970). The development of fibroblast colonies in monolayer cultures of Guinea-pig bone marrow and spleen cells. Cell Tissue Kinet.

[14] Dominici M, Le Blanc K, Mueller I, Slaper-Cortenbach I, Marini F, Krause D, Deans R, Keating A, Prockop D, Horwitz E (2006). Minimal criteria for defining multipotent mesenchymal stromal cells. The International Society for Cellular Therapy position statement. Cytotherapy..

[15] Djouad F, Bouffi C, Ghannam S, Noel D, Jorgensen C (2009). Mesenchymal stem cells: innovative therapeutic tools for rheumatic diseases. Nat Rev Rheumatol.

[16] Polymeri A, Giannobile WV, Kaigler D (2016). Bone marrow stromal stem cells in tissue engineering and regenerative medicine. Horm Metab Res.

[17] Nowak WN, Taha H, Kachamakova-Trojanowska N, Stepniewski J, Markiewicz JA, Kusienicka A, Szade K, Szade A, Bukowska-Strakova K, Hajduk K, Kloska D, Kopacz A, Grochot-Przeczek A, Barthenheier K, Cauvin C, Dulak J, Jozkowicz A (2018). Murine bone marrow mesenchymal stromal cells respond efficiently to oxidative stress despite the low level of heme oxygenases 1 and 2. Antioxid Redox Signal.

[18] Mok PL, Leong CF, Cheong SK (2013). Cellular mechanisms of emerging applications of mesenchymal stem cells. Malays J Pathol.

[19] Macrin D, Joseph JP, Pillai AA, Devi A (2017). Eminent sources of adult mesenchymal stem cells and their therapeutic imminence. Stem Cell Rev.

[20] Fraser JK, Wulur I, Alfonso Z, Hedrick MH (2006). Fat tissue: an underappreciated source of stem cells for biotechnology. Trends Biotechnol.

[21] Gimble JM, Katz AJ, Bunnell BA (2007). Adipose-derived stem cells for regenerative medicine. Circ Res.

[22] Semon JA, Maness C, Zhang X, Sharkey SA, Beuttler MM, Shah FS, Pandey AC, Gimble JM, Zhang S, Scruggs BA, Strong AL, Strong TA, Bunnell BA (2014). Comparison of human adult stem cells from adipose tissue and bone marrow in the treatment of experimental autoimmune encephalomyelitis. Stem Cell Res Ther.

[23] Zemel R, Bachmetov L, Ad-El D, Abraham A, Tur-Kaspa R (2009). Expression of liver-specific markers in naive adipose-derived mesenchymal stem cells. Liver Int.

[24] Liu L, Gao J, Yuan Y, Chang Q, Liao Y, Lu F (2013). Hypoxia preconditioned human adipose derived mesenchymal stem cells enhance angiogenic potential via secretion of increased VEGF and bFGF. Cell Biol Int.

[25] McIlhenny S, Zhang P, Tulenko T, Comeau J, Fernandez S, Policha A, Ferroni M, Faul E, Bagameri G, Shapiro I, DiMuzio P (2015). eNOS transfection of adipose-derived stem cells yields bioactive nitric oxide production and improved results in vascular tissue engineering. J Tissue Eng Regen Med.

[26] Zhang S, Dong Z, Peng Z, Lu F (2014). Anti-aging effect of adipose-derived stem cells in a mouse model of skin aging induced by D-galactose. PLoS One.

[27] Mohammadzadeh A, Pourfathollah AA, Shahrokhi S, Hashemi SM, Moradi SL, Soleimani M (2014). Immunomodulatory effects of adipose-derived mesenchymal stem cells on the gene expression of major transcription factors of T cell subsets. Int Immunopharmacol.

[28] Peng W, Gao T, Yang ZL, Zhang SC, Ren ML, Wang ZG, Zhang B (2012). Adipose-derived stem cells induced dendritic cells undergo tolerance and inhibit Th1 polarization. Cell Immunol.

[29] Sadick MD, Heinzel FP, Holaday BJ, Pu RT, Dawkins RS, Locksley RM (1990). Cure of murine leishmaniasis with anti-interleukin 4 monoclonal antibody. Evidence for a T cell-dependent, interferon gamma-independent mechanism. J Exp Med.

[30] Lafaille JJ, Keere FV, Hsu AL, Baron JL, Haas W, Raine CS, Tonegawa S (1997). Myelin basic protein-specific T helper 2 (Th2) cells cause experimental autoimmune encephalomyelitis in immunodeficient hosts rather than protect them from the disease. J Exp Med.

[31] Munoz MF, Arguelles S, Guzman-Chozas M, Guillen-Sanz R, Franco JM, Pintor-Toro JA, Cano M, Ayala A (2018). Cell tracking, survival, and differentiation capacity of adipose-derived stem cells after engraftment in rat tissue. J Cell Physiol.

[32] Pachon-Pena G, Yu G, Tucker A, Wu X, Vendrell J, Bunnell BA, Gimble JM (2011). Stromal stem cells from adipose tissue and bone marrow of age-matched female donors display distinct immunophenotypic profiles. J Cell Physiol.

[33] Riekstina U, Cakstina I, Parfejevs V, Hoogduijn M, Jankovskis G, Muiznieks I, Muceniece R, Ancans J (2009). Embryonic stem cell marker expression pattern in human mesenchymal stem cells derived from bone marrow, adipose tissue, heart and dermis. Stem Cell Rev.

[34] Siegel G, Kluba T, Hermanutz-Klein U, Bieback K, Northoff H, Schafer R (2013). Phenotype, donor age and gender affect function of human bone marrow-derived mesenchymal stromal cells. BMC Med.

[35] Ding DC, Chou HL, Hung WT, Liu HW, Chu TY (2013). Human adipose-derived stem cells cultured in keratinocyte serum free medium: Donor’s age does not affect the proliferation and differentiation capacities. J Biomed Sci.

[36] Sheykhhasan M, Qomi RT, Ghiasi M (2015). Fibrin scaffolds designing in order to human adipose-derived mesenchymal stem cells differentiation to chondrocytes in the presence of TGF-beta3. Int J Stem Cells.

[37] Banas A, Teratani T, Yamamoto Y, Tokuhara M, Takeshita F, Osaki M, Kawamata M, Kato T, Okochi H, Ochiya T (2008). IFATS collection: in vivo therapeutic potential of human adipose tissue mesenchymal stem cells after transplantation into mice with liver injury. Stem Cells.

[38] Hao T, Chen J, Zhi S, Zhang Q, Chen G, Yu F (2017). Comparison of bone marrow-vs. adipose tissue-derived mesenchymal stem cells for attenuating liver fibrosis. Exp Ther Med.

[39] Xu LJ, Wang SF, Wang DQ, Ma LJ, Chen Z, Chen QQ, Wang J, Yan L (2017). Adipose-derived stromal cells resemble bone marrow stromal cells in hepatocyte differentiation potential in vitro and in vivo. World J Gastroenterol.

[40] Talens-Visconti R, Bonora A, Jover R, Mirabet V, Carbonell F, Castell JV, Gomez-Lechon MJ (2006). Hepatogenic differentiation of human mesenchymal stem cells from adipose tissue in comparison with bone marrow mesenchymal stem cells. World J Gastroenterol.

[41] Zare H, Jamshidi S, Dehghan MM, Saheli M, Piryaei A (2018). Bone marrow or adipose tissue mesenchymal stem cells: comparison of the therapeutic potentials in mice model of acute liver failure. J Cell Biochem.

[42] Salomone F, Barbagallo I, Puzzo L, Piazza C, Li VG (2013). Efficacy of adipose tissue-mesenchymal stem cell transplantation in rats with acetaminophen liver injury. Stem Cell Res.

[43] Potdar P, Sutar J (2010). Establishment and molecular characterization of mesenchymal stem cell lines derived from human visceral & subcutaneous adipose tissues. J Stem Cells Regen Med.

[44] Huang YJ, Chen P, Lee CY, Yang SY, Lin MT, Lee HS, Wu YM (2016). Protection against acetaminophen-induced acute liver failure by omentum adipose tissue derived stem cells through the mediation of Nrf2 and cytochrome P450 expression. J Biomed Sci.

[45] Deng L, Kong X, Liu G, Li C, Chen H, Hong Z, Liu J, Xia J (2016). Transplantation of adipose-derived mesenchymal stem cells efficiently rescues thioacetamide-induced acute liver failure in mice. Transplant Proc.

[46] Tautenhahn HM, Bruckner S, Baumann S, Winkler S, Otto W, von Bergen M, Bartels M, Christ B (2016). Attenuation of postoperative acute liver failure by mesenchymal stem cell treatment due to metabolic implications. Ann Surg.

[47] Lee SW, Chong JU, Min SO, Bak SY, Kim KS (2017). Are adipose-derived stem cells from liver falciform ligaments another possible source of mesenchymal stem cells?. Cell Transplant.

[48] Gimble J, Guilak F (2003). Adipose-derived adult stem cells: isolation, characterization, and differentiation potential. Cytotherapy..

[49] Strong AL, Bowles AC, Wise RM, Morand JP, Dutreil MF, Gimble JM, Bunnell BA (2016). Human adipose stromal/stem cells from obese donors show reduced efficacy in halting disease progression in the experimental autoimmune encephalomyelitis model of multiple sclerosis. Stem Cells.

[50] Hu Chenxia, Zhou Ning, Li Jianzhou, Shi Ding, Cao Hongcui, Li Jun, Li Lanjuan (2016). Porcine Adipose-Derived Mesenchymal Stem Cells Retain Their Stem Cell Characteristics and Cell Activities While Enhancing the Expression of Liver-Specific Genes after Acute Liver Failure. International Journal of Molecular Sciences.

[51] Wang Y, Wang F, Zhao H, Zhang X, Chen H, Zhang K (2014). Human adipose-derived mesenchymal stem cells are resistant to HBV infection during differentiation into hepatocytes in vitro. Int J Mol Sci.

[52] Jin Y, Yang L, Zhang Y, Gao W, Yao Z, Song Y, Wang Y (2017). Effects of age on biological and functional characterization of adiposederived stem cells from patients with endstage liver disease. Mol Med Rep.

[53] Lee KS, Kang HW, Lee HT, Kim HJ, Kim CL, Song JY, Lee KW, Cha SH (2014). Sequential sub-passage decreases the differentiation potential of canine adipose-derived mesenchymal stem cells. Res Vet Sci.

[54] Wang X, Liu C, Li S, Xu Y, Chen P, Liu Y, Ding Q, Wahapu W, Hong B, Yang M (2015). Effects of continuous passage on immunomodulatory properties of human adipose-derived stem cells. Cell Tissue Bank.

[55] Meza-Zepeda LA, Noer A, Dahl JA, Micci F, Myklebost O, Collas P (2008). High-resolution analysis of genetic stability of human adipose tissue stem cells cultured to senescence. J Cell Mol Med.

[56] Liang L, Ma T, Chen W, Hu J, Bai X, Li J, Liang T (2009). Therapeutic potential and related signal pathway of adipose-derived stem cell transplantation for rat liver injury. Hepatol Res.

[57] Banas A, Teratani T, Yamamoto Y, Tokuhara M, Takeshita F, Quinn G, Okochi H, Ochiya T (2007). Adipose tissue-derived mesenchymal stem cells as a source of human hepatocytes. Hepatology..

[58] Winkler S, Hempel M, Bruckner S, Mallek F, Weise A, Liehr T, Tautenhahn HM, Bartels M, Christ B (2015). Mouse white adipose tissue-derived mesenchymal stem cells gain pericentral and periportal hepatocyte features after differentiation in vitro, which are preserved in vivo after hepatic transplantation. Acta Physiol (Oxf).

[59] Yamamoto Y, Banas A, Murata S, Ishikawa M, Lim CR, Teratani T, Hatada I, Matsubara K, Kato T, Ochiya T (2008). A comparative analysis of the transcriptome and signal pathways in hepatic differentiation of human adipose mesenchymal stem cells. FEBS J.

[60] Bonora-Centelles A, Jover R, Mirabet V, Lahoz A, Carbonell F, Castell JV, Gomez-Lechon MJ (2009). Sequential hepatogenic transdifferentiation of adipose tissue-derived stem cells: relevance of different extracellular signaling molecules, transcription factors involved, and expression of new key marker genes. Cell Transplant.

[61] Sun J, Yuan Y, Qin H, Ying C, Liu W, Zhang J, He Y, Liu Z (2013). Serum from hepatectomized rats induces the differentiation of adipose tissue mesenchymal stem cells into hepatocyte-like cells and upregulates the expression of hepatocyte growth factor and interleukin-6 in vitro. Int J Mol Med.

[62] Nhung TH, Nam NH, Nguyen NT, Nghia H, Van Thanh N, Ngoc PK, Van Pham P (2015). A comparison of the chemical and liver extract-induced hepatic differentiation of adipose derived stem cells. In Vitro Cell Dev Biol Anim.

[63] Alizadeh E, Eslaminejad MB, Akbarzadeh A, Sadeghi Z, Abasi M, Herizchi R, Zarghami N (2016). Upregulation of MiR-122 via trichostatin A treatments in hepatocyte-like cells derived from mesenchymal stem cells. Chem Biol Drug Des.

[64] Alizadeh E, Zarghami N, Eslaminejad MB, Akbarzadeh A, Barzegar A, Mohammadi SA (2016). The effect of dimethyl sulfoxide on hepatic differentiation of mesenchymal stem cells. Artif Cells Nanomed Biotechnol..

[65] Banas A, Teratani T, Yamamoto Y, Tokuhara M, Takeshita F, Osaki M, Kato T, Okochi H, Ochiya T (2009). Rapid hepatic fate specification of adipose-derived stem cells and their therapeutic potential for liver failure. J Gastroenterol Hepatol.

[66] Xu F, Liu J, Deng J, Chen X, Wang Y, Xu P, Cheng L, Fu Y, Cheng F, Yao Y, Zhang Y, Huang M, Yu D, Wei Y, Deng H (2015). Rapid and high-efficiency generation of mature functional hepatocyte-like cells from adipose-derived stem cells by a three-step protocol. Stem Cell Res Ther.

[67] Han SM, Coh YR, Ahn JO, Jang G, Yum SY, Kang SK, Lee HW, Youn HY (2015). Enhanced hepatogenic transdifferentiation of human adipose tissue mesenchymal stem cells by gene engineering with Oct4 and Sox2. PLoS One.

[68] Davoodian N, Lotfi AS, Soleimani M, Mowla SJ (2014). MicroRNA-122 overexpression promotes hepatic differentiation of human adipose tissue-derived stem cells. J Cell Biochem.

[69] Chen KD, Hsu LW, Goto S, Huang KT, Nakano T, Weng WT, Lai CY, Kuo YR, Chiu KW, Wang CC, Cheng YF, Lin CC, Ma YY, Chen CL (2014). Regulation of heme oxygenase 1 expression by miR-27b with stem cell therapy for liver regeneration in rats. Transplant Proc.

[70] Davoodian N, Lotfi AS, Soleimani M, Ghaneialvar H (2017). The combination of miR-122 overexpression and Let-7f silencing induces hepatic differentiation of adipose tissue-derived stem cells. Cell Biol Int.

[71] Ghaedi M, Tuleuova N, Zern MA, Wu J, Revzin A (2011). Bottom-up signaling from HGF-containing surfaces promotes hepatic differentiation of mesenchymal stem cells. Biochem Biophys Res Commun.

[72] Ghaderi Gandomani M, Sahebghadam Lotfi A, Kordi Tamandani D, Arjmand S, Alizadeh S (2017). The enhancement of differentiating adipose derived mesenchymal stem cells toward hepatocyte like cells using gelatin cryogel scaffold. Biochem Biophys Res Commun.

[73] Mohammadpour A, Arjmand S, Lotfi AS, Tavana H, Kabir-Salmani M (2018). Promoting hepatogenic differentiation of human mesenchymal stem cells using a novel laminin-containing gelatin cryogel scaffold. Biochem Biophys Res Commun.

[74] Zhang X, Dong J (2015). Direct comparison of different coating matrix on the hepatic differentiation from adipose-derived stem cells. Biochem Biophys Res Commun.

[75] Manzini BM, da Silva Santos Duarte A, Sankaramanivel S, Ramos AL, Latuf-Filho P, Escanhoela C, Kharmandayan P, Olalla Saad ST, Boin I, Malheiros Luzo AC (2015). Useful properties of undifferentiated mesenchymal stromal cells and adipose tissue as the source in liver-regenerative therapy studied in an animal model of severe acute fulminant hepatitis. Cytotherapy..

[76] Guo DL, Wang ZG, Xiong LK, Pan LY, Zhu Q, Yuan YF, Liu ZS (2017). Hepatogenic differentiation from human adipose-derived stem cells and application for mouse acute liver injury. Artif Cells Nanomed Biotechnol.

[77] Zhang S, Zhu Z, Wang Y, Liu S, Zhao C, Guan W, Zhao Y (2018). Therapeutic potential of Bama miniature pig adipose stem cells induced hepatocytes in a mouse model with acute liver failure. Cytotechnology..

[78] Yuan J, Li W, Huang J, Guo X, Li X, Lu X, Huang X, Zhang H (2015). Transplantation of human adipose stem cell-derived hepatocyte-like cells with restricted localization to liver using acellular amniotic membrane. Stem Cell Res Ther.

[79] Bruckner S, Zipprich A, Hempel M, Thonig A, Schwill F, Roderfeld M, Roeb E, Christ B (2017). Improvement of portal venous pressure in cirrhotic rat livers by systemic treatment with adipose tissue-derived mesenchymal stromal cells. Cytotherapy..

[80] Almalki SG, Agrawal DK (2016). Key transcription factors in the differentiation of mesenchymal stem cells. Differentiation..

[81] Saito Y, Shimada M, Utsunomiya T, Ikemoto T, Yamada S, Morine Y, Imura S, Mori H, Arakawa Y, Kanamoto M, Iwahashi S, Takasu C (2014). Homing effect of adipose-derived stem cells to the injured liver: the shift of stromal cell-derived factor 1 expressions. J Hepatobiliary Pancreat Sci.

[82] Zhang J, Zhou S, Zhou Y, Feng F, Wang Q, Zhu X, Ai H, Huang X, Zhang X (2014). Hepatocyte growth factor gene-modified adipose-derived mesenchymal stem cells ameliorate radiation induced liver damage in a rat model. PLoS One.

[83] Tang WP, Akahoshi T, Piao JS, Narahara S, Murata M, Kawano T, Hamano N, Ikeda T, Hashizume M (2015). Basic fibroblast growth factor-treated adipose tissue-derived mesenchymal stem cell infusion to ameliorate liver cirrhosis via paracrine hepatocyte growth factor. J Gastroenterol Hepatol.

[84] Liu T, Mu H, Shen Z, Song Z, Chen X, Wang Y (2016). Autologous adipose tissuederived mesenchymal stem cells are involved in rat liver regeneration following repeat partial hepatectomy. Mol Med Rep.

[85] Chen KD, Huang KT, Lin CC, Weng WT, Hsu LW, Goto S, Nakano T, Lai CY, Kung CP, Chiu KW, Wang CC, Cheng YF, Ma YY, Chen CL (2016). MicroRNA-27b enhances the hepatic regenerative properties of adipose-derived mesenchymal stem cells. Mol Ther Nucleic Acids.

[86] Kim DH, Je CM, Sin JY, Jung JS (2003). Effect of partial hepatectomy on in vivo engraftment after intravenous administration of human adipose tissue stromal cells in mouse. Microsurgery..

[87] Kim SJ, Park KC, Lee JU, Kim KJ, Kim DG (2011). Therapeutic potential of adipose tissue-derived stem cells for liver failure according to the transplantation routes. J Korean Surg Soc.

[88] Teshima T, Matsumoto H, Michishita M, Matsuoka A, Shiba M, Nagashima T, Koyama H (2017). Allogenic adipose tissue-derived mesenchymal stem cells ameliorate acute hepatic injury in dogs. Stem Cells Int.

[89] Wang Y, Lian F, Li J, Fan W, Xu H, Yang X, Liang L, Chen W, Yang J (2012). Adipose derived mesenchymal stem cells transplantation via portal vein improves microcirculation and ameliorates liver fibrosis induced by CCl4 in rats. J Transl Med.

[90] Wan CD, Cheng R, Wang HB, Liu T (2008). Immunomodulatory effects of mesenchymal stem cells derived from adipose tissues in a rat orthotopic liver transplantation model. Hepatobiliary Pancreat Dis Int.

[91] Saidi RF, Rajeshkumar B, Shariftabrizi A, Bogdanov AA, Zheng S, Dresser K, Walter O (2014). Human adipose-derived mesenchymal stem cells attenuate liver ischemia-reperfusion injury and promote liver regeneration. Surgery..

[92] Lam PK, Chong CCN, Lo AWI, Chan AWH, Tong CSW, Chin DWC, Wong KHK, Choy RKW, Fung AK, Wang YX, To KF, Lai PBS (2017). Topical application of mesenchymal stromal cells ameliorated liver parenchyma damage after ischemia-reperfusion injury in an animal model. Transplant Direct.

[93] Ge Y, Zhang Q, Li H, Bai G, Jiao Z, Wang H (2018). Adipose-derived stem cells alleviate liver apoptosis induced by ischemia-reperfusion and laparoscopic hepatectomy in swine. Sci Rep.

[94] Ge Y, Zhang Q, Jiao Z, Li H, Bai G, Wang H (2018). Adipose-derived stem cells reduce liver oxidative stress and autophagy induced by ischemia-reperfusion and hepatectomy injury in swine. Life Sci.

[95] Sun CK, Chang CL, Lin YC, Kao YH, Chang LT, Yen CH, Shao PL, Chen CH, Leu S, Yip HK (2012). Systemic administration of autologous adipose-derived mesenchymal stem cells alleviates hepatic ischemia-reperfusion injury in rats. Crit Care Med.

[96] Gu X, Manautou JE (2012). Molecular mechanisms underlying chemical liver injury. Expert Rev Mol Med.

[97] Saidi R, Rajeshkumar R, Shariftabrizi A, Zimmerman A, Walter O (2015). Human adipose-derived mesenchymal stem cells promote liver regeneration. J Investig Surg.

[98] Zhang S, Liu P, Chen L, Wang Y, Wang Z, Zhang B (2015). The effects of spheroid formation of adipose-derived stem cells in a microgravity bioreactor on stemness properties and therapeutic potential. Biomaterials..

[99] Xu L, Wang S, Sui X, Wang Y, Su Y, Huang L, Zhang Y, Chen Z, Chen Q, Du H, Yan L (2017). Mesenchymal stem cell-seeded regenerated silk fibroin complex matrices for liver regeneration in an animal model of acute liver failure. ACS Appl Mater Interfaces.

[100] Kubo N, Narumi S, Kijima H, Mizukami H, Yagihashi S, Hakamada K, Nakane A (2012). Efficacy of adipose tissue-derived mesenchymal stem cells for fulminant hepatitis in mice induced by concanavalin A. J Gastroenterol Hepatol.

[101] Higashimoto M, Sakai Y, Takamura M, Usui S, Nasti A, Yoshida K, Seki A, Komura T, Honda M, Wada T, Furuichi K, Ochiya T, Kaneko S (2013). Adipose tissue derived stromal stem cell therapy in murine ConA-derived hepatitis is dependent on myeloid-lineage and CD4+ T-cell suppression. Eur J Immunol.

[102] Yoshizumi Y, Yukawa H, Iwaki R, Fujinaka S, Kanou A, Kanou Y, Yamada T, Nakagawa S, Ohara T, Nakagiri K, Ogihara Y, Tsutsui Y, Hayashi Y, Ishigami M, Baba Y, Ishikawa T (2017). Immunomodulatory effects of adipose tissue-derived stem cells on concanavalin A-induced acute liver injury in mice. Cell Med.

[103] Li M, Lv Y, Chen F, Wang X, Zhu J, Li H, Xiao J (2018). Co-stimulation of LPAR1 and S1PR1/3 increases the transplantation efficacy of human mesenchymal stem cells in drug-induced and alcoholic liver diseases. Stem Cell Res Ther.

[104] Liu Y, Xiong Y, Xing F, Gao H, Wang X, He L, Ren C, Liu L, So KF, Xiao J (2017). Precise regulation of miR-210 is critical for the cellular homeostasis maintenance and transplantation efficacy enhancement of mesenchymal stem cells in acute liver failure therapy. Cell Transplant.

[105] Okura H, Soeda M, Morita M, Fujita M, Naba K, Ito C, Ichinose A, Matsuyama A (2015). Therapeutic potential of human adipose tissue-derived multi-lineage progenitor cells in liver fibrosis. Biochem Biophys Res Commun.

[106] Harn HJ, Lin SZ, Hung SH, Subeq YM, Li YS, Syu WS, Ding DC, Lee RP, Hsieh DK, Lin PC, Chiou TW (2012). Adipose-derived stem cells can abrogate chemical-induced liver fibrosis and facilitate recovery of liver function. Cell Transplant.

[107] Yu F, Ji S, Su L, Wan L, Zhang S, Dai C, Wang Y, Fu J, Zhang Q (2015). Adipose-derived mesenchymal stem cells inhibit activation of hepatic stellate cells in vitro and ameliorate rat liver fibrosis in vivo. J Formos Med Assoc.

[108] Sakai Y, Takamura M, Seki A, Sunagozaka H, Terashima T, Komura T, Yamato M, Miyazawa M, Kawaguchi K, Nasti A, Mochida H, Usui S, Otani N, Ochiya T, Wada T, Honda M, Kaneko S (2017). Phase I clinical study of liver regenerative therapy for cirrhosis by intrahepatic arterial infusion of freshly isolated autologous adipose tissue-derived stromal/stem (regenerative) cell. Regen Ther.

[109] Tang WP, Akahoshi T, Piao JS, Narahara S, Murata M, Kawano T, Hamano N, Ikeda T, Hashizume M (2016). Splenectomy enhances the therapeutic effect of adipose tissue-derived mesenchymal stem cell infusion on cirrhosis rats. Liver Int.

[110] Baig MT, Ali G, Awan SJ, Shehzad U, Mehmood A, Mohsin S, Khan SN, Riazuddin S (2017). Serum from CCl4-induced acute rat injury model induces differentiation of ADSCs towards hepatic cells and reduces liver fibrosis. Growth Factors.

[111] Kang H, Seo E, Park JM, Han NY, Lee H, Jun HS (2018). Effects of FGF21-secreting adipose-derived stem cells in thioacetamide-induced hepatic fibrosis. J Cell Mol Med.

[112] Lou G, Yang Y, Liu F, Ye B, Chen Z, Zheng M, Liu Y (2017). MiR-122 modification enhances the therapeutic efficacy of adipose tissue-derived mesenchymal stem cells against liver fibrosis. J Cell Mol Med.

